# Breast Cancer Surgical Specimens: A Marking Challenge and a Novel Solution—A Prospective, Randomized Study

**DOI:** 10.3390/biomedicines13040984

**Published:** 2025-04-17

**Authors:** András Drozgyik, Noémi Kránitz, Tamás Szabó, Dániel Kollár, István Á. Harmati, Renáta Rajnai, Tamás F. Molnár

**Affiliations:** 1 Department of Burns and Plastic Surgery, Petz Aladár University Teaching Hospital, 9024 Győr, Hungary; 2 Doctoral School of Clinical Sciences, University of Pécs Medical School, 7624 Pécs, Hungary; tfmolnar@gmail.com; 3 Department of Pathology, Petz Aladár University Teaching Hospital, 9024 Győr, Hungary; 4 Kirurgkliniken, Värnamo Sjukhus, 331 56 Värnamo, Sweden; 5 Department of Mathematics and Computational Sciences, Széchenyi István University, 9026 Győr, Hungary; harmati@sze.hu (I.Á.H.);; 6 Department of Operational Medicine, University of Pécs Medical School, 7624 Pécs, Hungary; 7 Department Vascular and Thoracic Surgery, Petz Aladár University Teaching Hospital, 9026 Győr, Hungary

**Keywords:** breast cancer, specimen orientation, surgical accuracy, pathology

## Abstract

**Background:** Accurate orientation of resected breast specimens is essential for proper pathological evaluation and margin assessment. Misorientation may compromise analysis, lead to imprecise re-excisions, and increase the risk of local recurrence. This study aims to evaluate a novel specimen plate designed to maintain consistent tissue orientation and compares its effectiveness to traditional suture marking. **Methods:** In a single-center, prospective, randomized two-arm trial, 56 specimens were oriented with the new plate and 54 with conventional sutures. Outcomes included intraoperative imaging interpretation, specimen handling, and pathological assessment, with a focus on orientation accuracy and margin evaluation. **Results:** The specimen plate significantly reduced misorientation (*p* < 0.01) and improved interpretation during intraoperative imaging. Pathologists reported greater ease in identifying direction and tumor-free zones, leading to a more accurate margin assessment. Non-R0 resections requiring re-excision were fewer with the specimen plate (8.9%) compared to suture marking (22.2%). **Conclusions:** The newly developed specimen plate can offer a reliable solution for improving specimen orientation in breast cancer surgery; however, further validation in multicenter studies is needed to confirm its applicability across diverse surgical settings. By ensuring consistent orientation and enhancing diagnostic interpretation, it may help reduce re-excisions and improve patient safety.

## 1. Introduction

Increased detection of non-palpable breast tumors emphasizes the need for precise preoperative marking and accurate specimen orientation for effective evaluation [1]. Accurate specimen orientation during breast cancer surgery is crucial for proper pathological evaluation and ensuring complete tumor resection [2].

When the margin status is uncertain, targeted re-excisions are often preferred over routine cavity shaving [3]. This approach allows for a more localized and directional removal of tissue in the affected area while preserving radiologically adequate margins. As a result, the total excised volume may remain equal to or even less than with circumferential cavity shaving, thereby achieving oncological safety with better cosmetic outcomes.

Intraoperative misorientation of resected tissue can lead to errors in margin assessment, potentially misorienting re-excisions, and increasing the risk of a local recurrence. Traditionally, surgeons rely on suture or clip marking techniques to orient specimens by indicating anatomical directions [4]. However, these methods are prone to variability, primarily due to the mobility and torsion of excised breast tissue during surgery and transportation to pathology.

**Figure 1:**
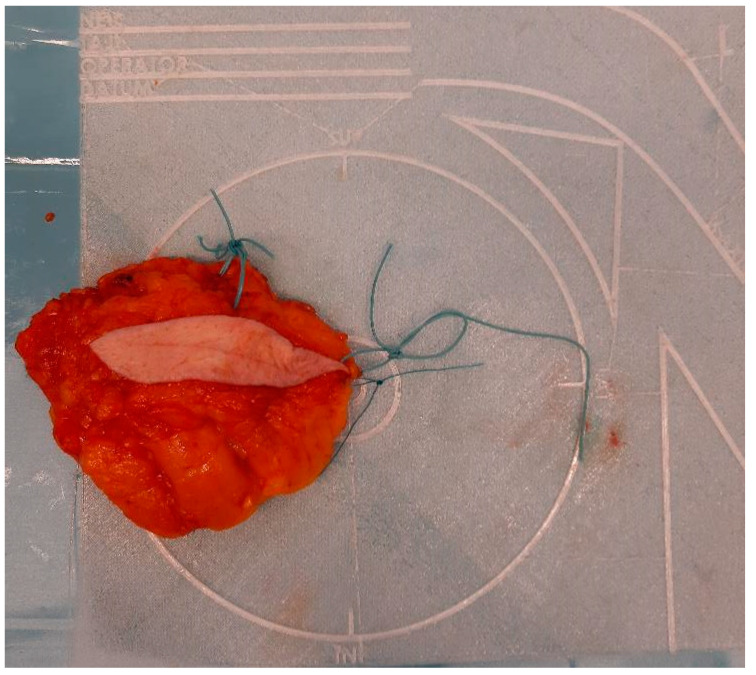
Properly oriented breast tissue specimen placed on the specimen plate used during the radio-guided removal of an occult lesion. This form of presentation allows the intuitive evaluation of the mammogram in the coronal plane and optimizes slicing for pathological processing.

To address the limitations of current practices, a novel specimen plate was previously developed and introduced to mitigate [5] the risk of misorientation (Figure 1).

The specimen plate design was based on the feedback from a nationwide, structured, formal survey conducted via an online questionnaire among Hungarian breast cancer centers. This survey systematically assessed current challenges in specimen orientation, the limitations of traditional marking techniques, and the specific needs of surgeons and pathologists. The collected data provided objective insights into the variability in orientation practices and the demand for a standardized, reproducible method to improve pathological accuracy [5].

**Figure 2:**
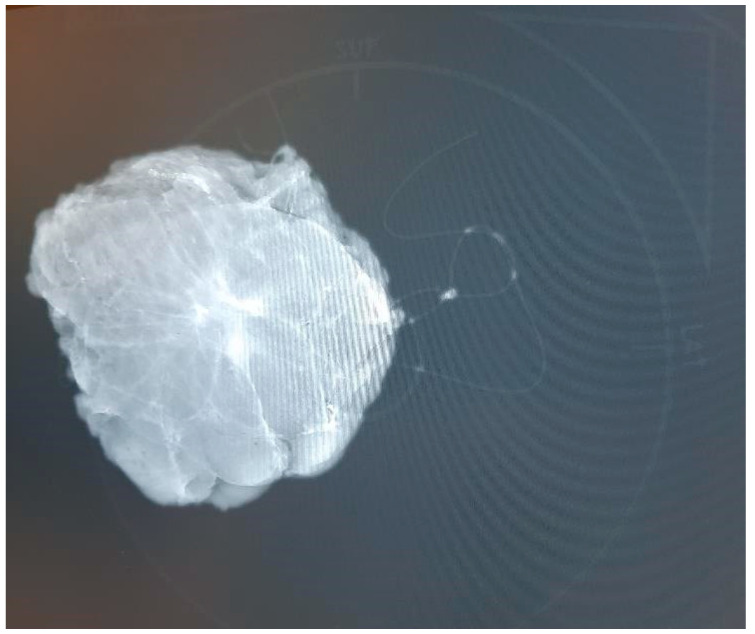
A mammographic image of the oriented breast tissue specimen with radio-guided tumor localization on the specimen plate. The specimen plate provides high-fidelity localization, accurately reflecting the specimen’s position within the breast. Traditional suture marking (single, long = lateral; double, short = superior) is also applied, using a thick yarn that creates an X-ray shadow.

Based on survey data, we developed a specimen plate that securely anchors excised breast tissue on a reproducible coronal plane, thereby enhancing orientation accuracy for both surgeons and pathologists. Additionally, the breast scheme on the plate is clearly visible in the frontal plane, facilitating the visualization of the excised tissue and tumor’s in vivo position in this orientation. The plate enables specimen mammography without causing a disruptive X-ray shadow, as the material is radiolucent, and its physical properties are detailed in Appendix A. This ensures that the plate does not interfere with standard imaging orientations. Radiologists typically rely on craniocaudal (CC) and mediolateral oblique (MLO) views when assessing breast lesions in mammographic imaging. However, surgeons usually conceptualize the tumor’s position within the breast in the coronal or frontal plane, which aligns more closely with their intraoperative perspective. A coronal plane image, supplemented by the breast scheme on the specimen plate, not only aids in avoiding misinterpretations but also enhances precision in margin assessment. By integrating this additional imaging perspective, the specimen plate facilitates an unambiguous understanding of the tumor’s in vivo location and improves the accuracy of the pathological evaluation. (Figure 2).

Despite its theoretical promise, the efficacy and real value of the specimen plate relative to traditional suture marking techniques required objective comparative investigation. A single-center, prospective, randomized comparative study was conducted to evaluate the scalable differences between traditional suture marking and the novel specimen plate in orienting the resected breast tissue.

## 2. Statistical Analyses

Python 3.11.5 (Python Software Foundation, Wilmington, DE, USA), Pandas 2.0.3 (pandas development team, open-source project, USA), SciPy 1.11.1 (SciPy community, open-source project, USA), Seaborn 0.12.2 (Michael Waskom, Stanford University, CA, USA), Matplotlib 3.7.2 (Matplotlib development team, open-source project, USA). The statistical methods included the z-test (u-test) for proportions, with results deemed significant if *p* < 0.05. For contingency table analysis, Chi-squared tests, a Fisher’s exact test, and its generalization, the Fisher–Freeman–Halton test, were applied as appropriate. Additionally, confidence intervals were calculated using multiple methods, including normal approximation, Agresti–Coull, beta, Wilson, and binomial tests.

## 3. Patients and Methods

### 3.1. Study Design

A single-center, prospective, randomized, non-blinded comparative interventional study was conducted in the Departments of Pathology and Surgery in our high-volume university hospital cancer center.

### 3.2. Patient Selection and Randomization

A total of 110 consecutive female patients, aged 29 to 86 years (mean age: 60.63 ± 11.82 years), diagnosed with malignant breast lesions and undergoing breast-conserving surgery, were enrolled in the study. Patients were randomly assigned into two groups. Randomization was carried out using a random number generator. A total of 56 patients were allocated into the Specimen Plate group, while the traditional suture marking group consisted of 54 patients. Specimens in the latter group were transported to the pathology department in standard pathological specimen containers. Patient safety was maintained by additional (standard) suture markings in the Specimen Plate group, meaning that in this group both techniques were used simultaneously.

### 3.3. Ages of Patients in the Examined Groups at the Time of the Operation

**Figure 3:**
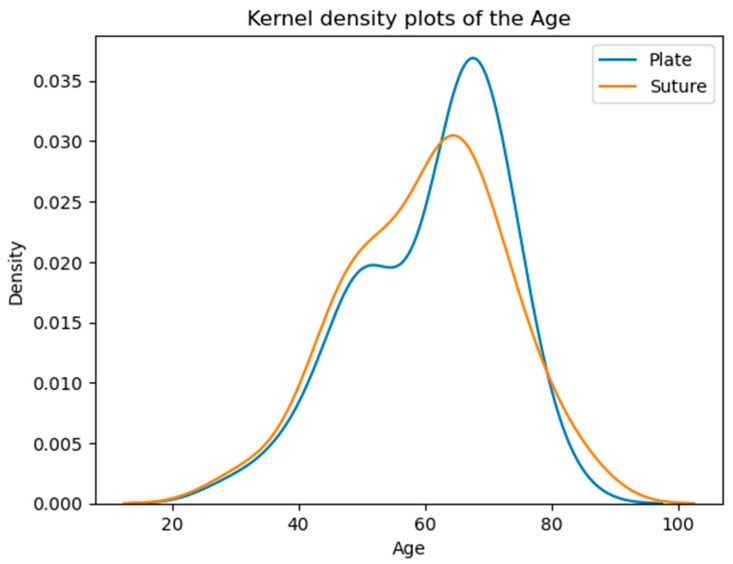
Age distribution of the patients. According to the Kolmogorov–Smirnov test, no significant difference is detected (*p* = 0.5373) between the two distributions. The vertical axis shows the estimated probability density of the age variable based on kernel density estimation.

The majority of patients fall within the 50–70 age range, with a noticeable peak around the 60s. The overall similarity in average and median ages suggests comparable age demographics across both groups (Table 1, Figure 3).

### 3.4. Body Mass Index (BMI) of Patients

**Figure 4:**
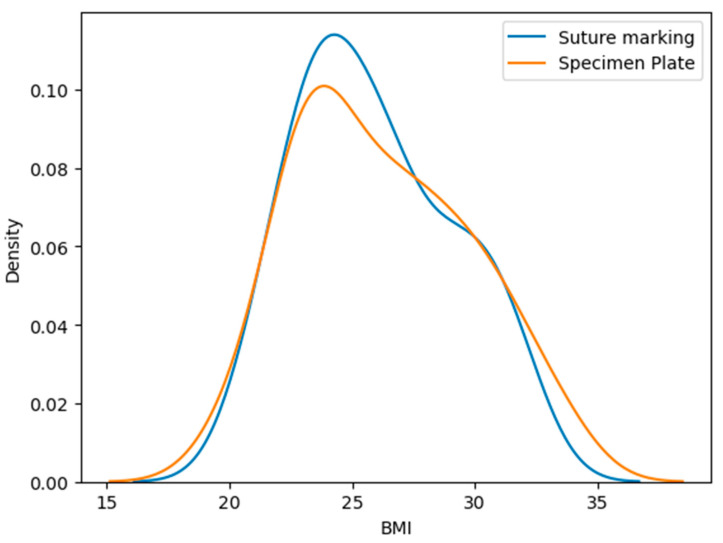
Distribution of body mass index (BMI) values in the Specimen Plate and Suture Marking groups. No significant difference was observed between the groups, supporting baseline comparability in terms of body habitus.

The mean BMI in the Specimen Plate group was 26.23 ± 3.55 kg/m^2^, while the Suture Marking group had a mean BMI of 25.96 ± 3.17 kg/m^2^, *p* = 0.9888 (Table 2, Figure 4). No statistically significant difference was observed in BMI distribution. The Kolmogorov–Smirnov test shows a significant difference. These findings reinforce the validity of group comparability.

### 3.5. Histological Types of Breast Cancer in the Study Groups

**Figure 5:**
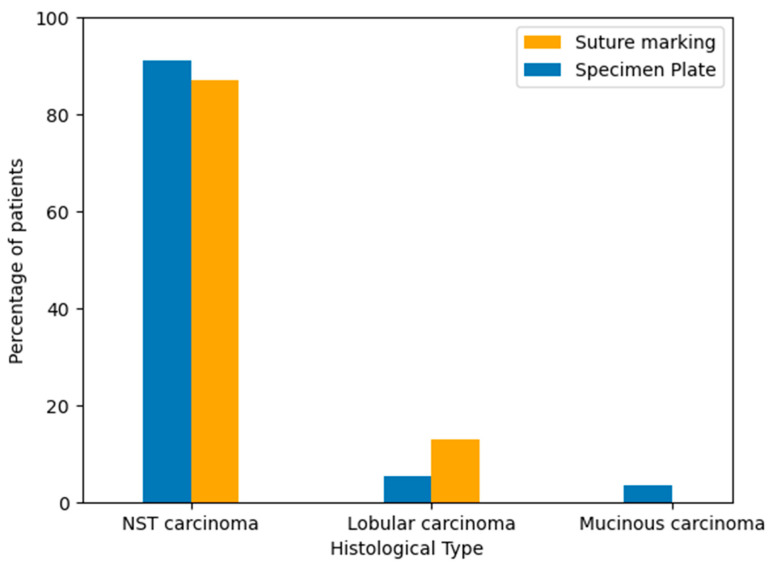
Distribution of breast cancer histological subtypes in the Specimen Plate and Suture Marking groups. NST carcinoma was the predominant type in both cohorts (*p* = 0.1734).

To evaluate the distribution of tumor histology between the two groups, breast cancer types were classified based on standard pathological diagnosis after core biopsy. The majority of cases in both groups were invasive carcinoma of no special type (NST). Lobular and mucinous carcinomas occurred in a minority of patients. The histological type distribution was similar between the Specimen Plate and Suture Marking groups, supporting cohort comparability (Table 3, Figure 5).

### 3.6. Immunohistochemical Subtypes of Breast Cancer

**Figure 6:**
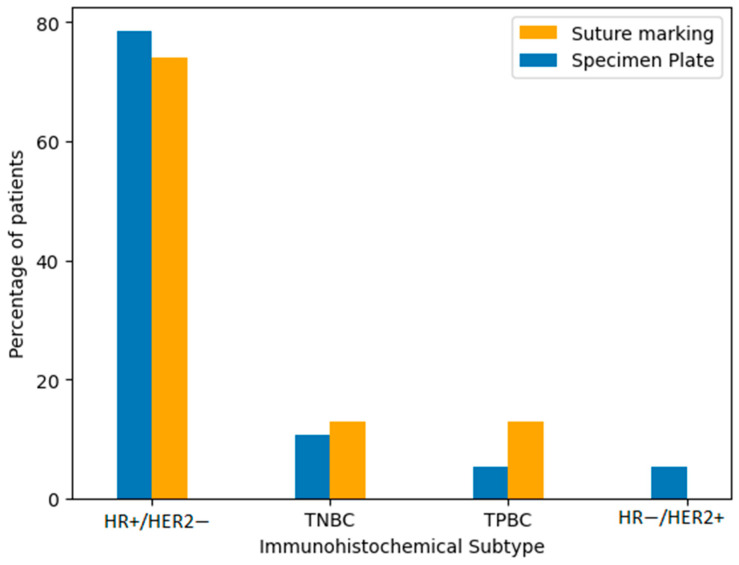
Distribution of immunohistochemical subtypes of breast cancer in the Specimen Plate and Suture Marking groups. HR+/HER2− tumors were the most common subtype in both cohorts (*p* = 0.2208).

Immunohistochemical classification included the following subtypes: hormone receptor-positive/HER2-negative (HR+/HER2−), triple-negative breast cancer (TNBC), triple-positive breast cancer (TPBC), and hormone receptor-negative/HER2-positive breast cancer (HR−/HER2+). The majority of tumors in both groups were HR+/HER2−. To verify the similarity in distribution, we performed comparative statistical analysis using both a Fisher’s exact test and a Chi-squared test. The *p*-value was 0.2208, indicating no statistically significant difference in subtype distribution between the Specimen Plate and Suture Marking groups (Table 4, Figure 6). These results confirm that the biological characteristics of the tumors were well balanced across both study arms.

To assess hormone receptor status, immunohistochemical staining was performed using antibodies against the estrogen receptor (ER) and progesterone receptor (PR). Tumors were considered hormone receptor-positive if the Allred score was 3 or higher, in accordance with international guidelines. For the sake of clarity, all such cases are referred to in this manuscript as hormone receptor-positive breast cancers.

### 3.7. Tumor Localization Within the Breast

**Figure 7:**
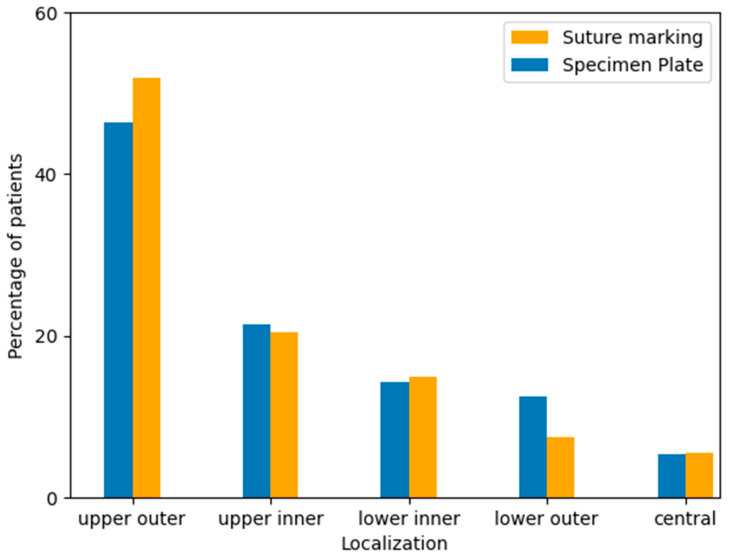
Distribution of tumor localization within the breast by quadrant in the Specimen Plate and Suture Marking groups. The upper outer quadrant was the most frequent tumor location in both cohorts, with similar distributions observed across all regions (*p* = 0.6577).

Tumor localization was recorded based on preoperative imaging and intraoperative findings, categorized by anatomical quadrants: upper outer, upper inner, lower inner, lower outer, and central regions. The majority of tumors in both groups were located in the upper outer quadrant, consistent with known epidemiological trends. No significant difference in tumor localization was observed between the two study groups (Table 5, Figure 7).

### 3.8. Tumor Proliferative Activity Based on Ki-67 Index

Tumor proliferation was assessed using the Ki-67 index, and tumors were categorized into low and high proliferative subgroups. In the suture marking group, 49 tumors (90.7%) were classified as low proliferation and 5 tumors (9.3%) as high proliferation. In the specimen plate group, 51 tumors (91.1%) were low proliferation and 5 tumors (8.9%) were high proliferation. Both the Fisher’s exact test and the chi-squared test yielded a *p*-value of 1.000, indicating no significant difference in proliferative activity between the two groups (Table 6).

### 3.9. Specimen Orientation Assessment

Specimen orientation was assessed based on predefined criteria, including clarity of orientation upon arrival at pathology, tumor localization within the specimen (both macroscopically and based on the mammogram), and side-specific clarity.

### 3.10. Criteria for Assessing Specimen Orientation

Specimen orientation and tumor localization were evaluated by the pathologist based on predefined binary (yes/no) questions, as outlined below:

**A:** Was the specimen’s orientation preserved unambiguously upon arrival in the pathology department?
**B:** Was tumor localization unambiguously identifiable on the mammogram?
**C:** Were the anatomical sides of the specimen (e.g., medial/lateral or superior/inferior) distinguishable?
**D:** Was tumor localization evident on macroscopic examination?
**E:** Was neoadjuvant therapy performed?
**F:** Was there complete radiologic or pathologic regression, or no regression?
**G:** Was there any ambiguity in distinguishing between opposing sides of the specimen (e.g., due to similar suture lengths or indistinct markers), such that the specimen could potentially be misoriented or rotated 180 degrees?

## 4. Results

### 4.1. Specimen Orientation and Tumor Localization

The results of the comparative analysis are summarized as follows:

**A:** Orientation clarity upon arrival at pathology was comparable between the two groups (*p* = 0.0737).
**B**: Mammographic clarity of tumor localization was significantly less evident in the Suture Marking Group (*p* = 0.0001).
**C:** Clarity of specimen laterality (left/right) was not significantly different (*p* = 0.1461).
**D:** Macroscopic clarity of tumor localization within the specimen showed no significant difference (*p* = 0.5834).
**E:** Neoadjuvant therapy usage was similar between the groups (*p* = 0.7555).
**F:** Complete radiologic or pathologic regression rates showed no significant difference (*p* = 0.5252).
**G:** Potential for 180-degree rotation of the specimen was identical across all cases in both groups.

**Figure 8:**
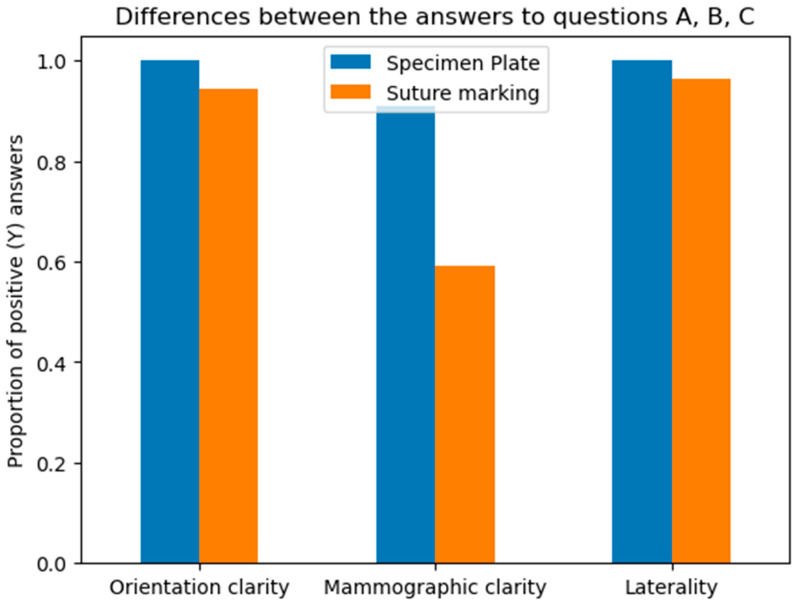
Differences in responses to questions A (Orientation clarity), B (Mammographic clarity) and C (Clarity of specimen laterality), comparing the specimen plate technique to traditional suture marking. Mammographic clarity showed strong significance in favor of the Specimen Plate Group. The vertical axis indicates the proportion of positive responses for each question in both groups.

**Figure 9:**
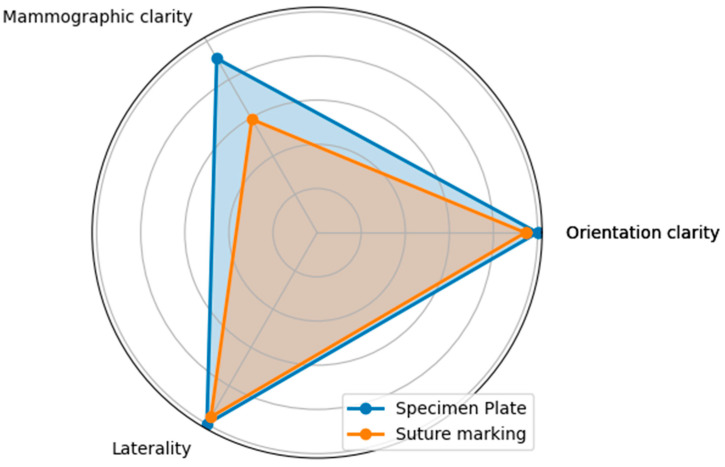
The radar diagram compares the performance of the Specimen Plate and traditional Suture Marking techniques across key parameters related to breast specimen orientation (Questions A—Orientation clarity, B—Mammographic clarity, and C—Clarity of specimen laterality). The shaded areas highlight the differences in performance between the two methods.

Questions A, B, and C focused on the differences between the specimen plate and the traditional method, specifically evaluating whether the orientation of the breast specimen was improved when using the specimen plate. The results show that the specimen plate provided more accurate orientation compared to the traditional method (Figure 8 and Figure 9).

**Figure 10:**
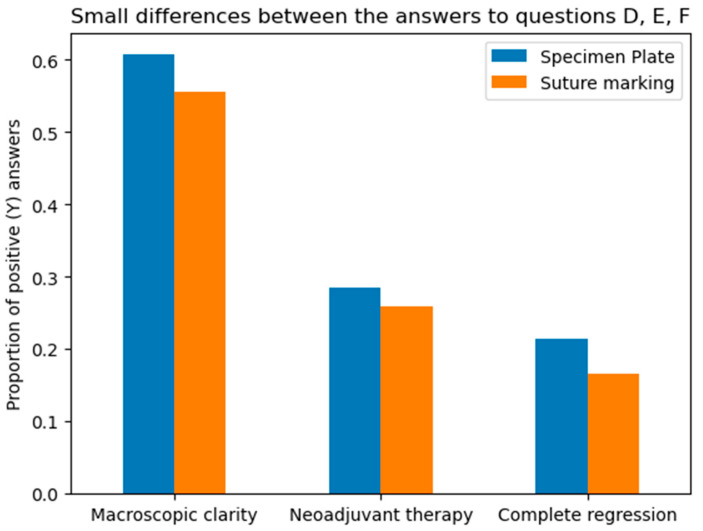
Differences between the Specimen Plate and traditional Suture Marking methods in responses to Questions D, E, and F. (Question D—macroscopic clarity of tumor localization, E—neoadjuvant therapy usage, and F—complete radiologic or pathologic regression). While small variations exist, none of the differences for these parameters were statistically significant, indicating comparable numbers of the two methods for the evaluated factors. The vertical axis indicates the proportion of positive (“Yes”) responses for each question in both groups.

**Figure 11:**
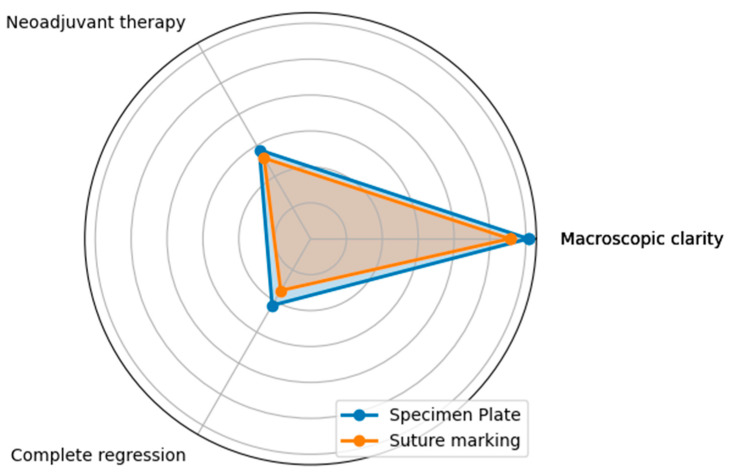
The radar diagram visualizes the performance of the Specimen Plate and traditional Suture Marking techniques for Questions D, E, and F, highlighting their similarities. The overlapping areas in the radar plot emphasize the minimal differences between the two groups for these parameters, reinforcing their comparable results in these aspects.

Questions D, E, and F assessed whether the two groups in the study were properly randomized and ensured there were no statistical differences between the groups that could bias the results. (Figure 10 and Figure 11).

The macroscopic clarity of tumor localization results underscores the proper randomization of the two groups, as they demonstrate that the percentage of clinically (in vivo) non-palpable or poorly palpable tumors became macroscopically identifiable for the pathologist after resection.

The rates of neoadjuvant therapy administration (Question E) and the proportions of complete or absent tumor regression (Question F) were also similar across the two groups, with no statistically significant differences (*p* = 0.7555 and *p* = 0.5252, respectively).

The lack of significant differences in tumor palpability after resection, neoadjuvant therapy usage, regression patterns, and BMI between the two arms confirms the internal validity of our comparative analysis. Additionally, the two groups showed similar distributions in patient age, tumor localization within the breast, histological subtypes, and immunohistochemical profiles.

### 4.2. Pathological Tumor (pT) Stage Distribution

**Figure 12:**
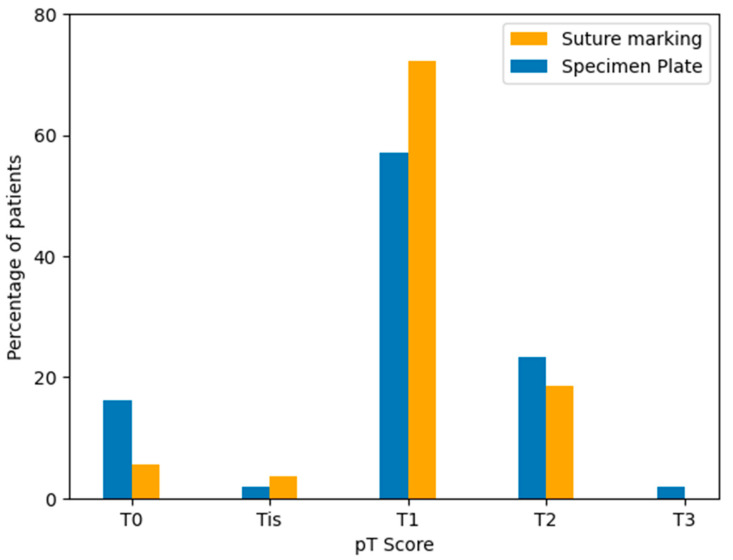
Distribution of pathological tumor stages (pT) in the Specimen Plate and Suture Marking groups. Most tumors were classified as pT1 in both cohorts. A higher proportion of pT0 tumors was observed in the Specimen Plate group, corresponding to neoadjuvant therapy-associated regression.

Postoperative pathological tumor staging (pT) was assessed for all cases according to the TNM classification system. The majority of tumors in both groups were staged as pT1, followed by pT2 and pT0 cases. Although the Specimen Plate group showed a higher proportion of pT0 tumors, indicating a trend toward better response to neoadjuvant therapy, the overall distribution of pT categories did not differ significantly between the two groups (Table 7, Figure 12).

### 4.3. Orientation Accuracy and Reliability—Comparison of Specimen Plate and Suture Marking Methods

**Figure 13:**
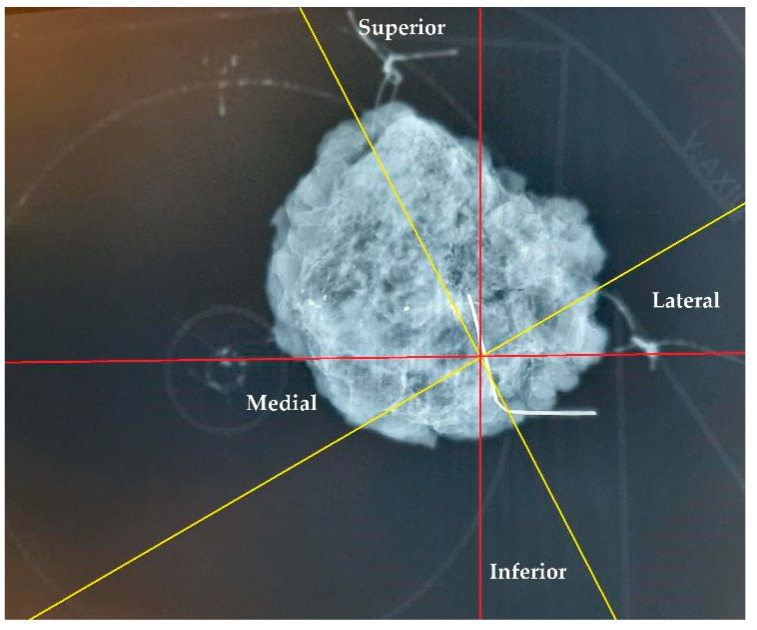
Orientation of a breast specimen using both suture marking and the specimen plate. The coronally positioned specimen is secured on the specimen plate, with additional suture markings: a longer thread marking on the lateral side and double shorter threads on the cranial side. A deviation of 30° is observed when comparing suture markings with the specimen plate orientation. The yellow lines represent the potential superior–inferior and lateral–medial axes based on suture markings, while the red lines denote the actual orientations established using the specimen plate.

The specimen plate design offers enhanced stabilization of the excised breast tissue compared to traditional suture marking. Rather than relying solely on sutures attached to sides of the tissue for orientation, the plate secures the tissue at its basal surface, thereby reducing torsion and mobility and ensuring more accurate preservation of the anatomical localization. In cases where both the specimen plate and suture markings were used (n = 56), 11 cases (19.64%) demonstrated an angular deviation of ≥20° between the two methods. This discrepancy highlights the potential for misleading pathological evaluations and incorrect re-excision directions, especially when relying solely on text descriptions. As demonstrated in Figure 13, a comparison of suture markings with specimen plate orientations revealed angular deviations of up to 30° (even with accurate suture markings, due to tissue torsion), underscoring the limitations of suture markings alone.

**Figure 14:**
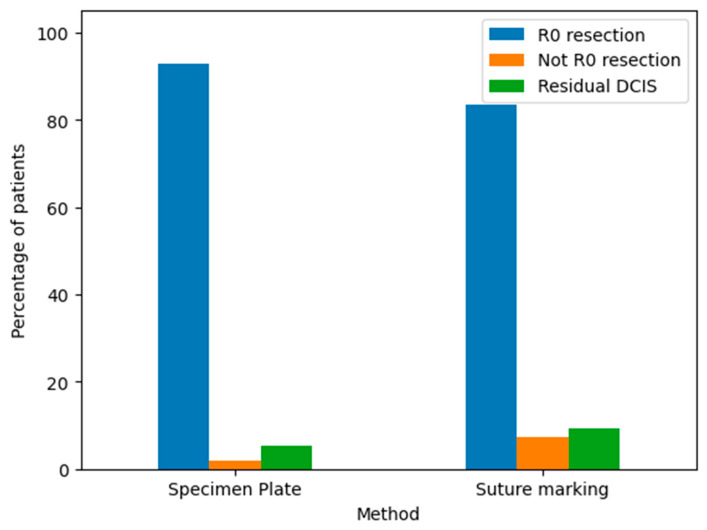
Pathological findings of resected breast specimens for the Specimen Plate and Suture Marking methods. The results are categorized as follows: R0 resection: Indicates a complete tumor removal with negative margins. This was the most common outcome for both methods. Not R0 resection: Indicates incomplete tumor removal with positive margins. This was infrequent for both techniques. Residual DCIS: Indicates the presence of residual ductal carcinoma in situ. The frequency of this outcome was comparable between the two groups.

The pathological outcomes of resected breast specimens using the Specimen Plate and Suture Marking methods are illustrated in the corresponding bar chart (Figure 14).

Both the Specimen Plate and Suture Marking methods predominantly achieved R0 resections, with minimal differences in pathological outcomes, underscoring their similar effectiveness in ensuring clear surgical margins. However, a detailed comparison (Table 8) reveals notable differences in orientation reliability and tumor localization. All specimens in the Specimen Plate Group maintained their intended orientation upon arrival at pathology, with no instances of 180-degree rotation. In contrast, the Suture Marking Group exhibited slightly lower orientation reliability (96.3%). Furthermore, the Specimen Plate Group demonstrated superior performance in macroscopic tumor localization (60.71% vs. 44.44%) and mammographic orientation (80.4% vs. 13%). Detailed results are presented in Table 8.

### 4.4. R0 Resection Rates by Immunohistochemical Subtype in the Specimen Plate Group

Distribution of R0 and non-R0 resections across immunohistochemical subtypes is shown in Table 9 (Specimen Plate Group) and Table 10 (Suture Marking Group). In both groups, the majority of non-R0 resections occurred in HR+/HER2− tumors. No non-R0 resections were observed in TPBC or HR−/HER2+ subtypes.

### 4.5. R0 Resection Rates by Immunohistochemical Subtype in the Suture Marking Group

The immunohistochemical subtypes were defined as follows: HR+/HER2− indicates hormone receptor-positive, HER2-negative tumors; TNBC refers to triple-negative breast cancer (negative for estrogen receptor, progesterone receptor, and HER2); TPBC refers to triple-positive tumors (positive for both hormone receptors and HER2); and HR−/HER2+ includes tumors that are hormone receptor-negative and HER2-positive.

## 5. Discussion

Current studies indicate that re-excision rates after breast-conserving surgery vary significantly, reaching up to 30%, with many re-operations performed even when margins are negative [6,7,8]. Advances in oncoplastic techniques and neoadjuvant treatments have reduced these rates, but challenges remain [6,9]. Margin assessment depends on multiple factors, including pathological sectioning techniques, tumor characteristics, and specimen handling. The irregular edges of tumors and compression of specimens during mammography further complicate accurate margin evaluation [10]. Although consensus guidelines recommend a negative margin of ‘no tumor on ink’ to achieve R0 resection, practice variability remains widespread, highlighting the importance of establishing standardized approaches [11].

Increased detection of non-palpable tumors has underscored the importance of precise preoperative marking and intraoperative radiological evaluation. Techniques like wire-guided and radio-guided localization are widely used, with emerging promising technologies such as magnetic and radar localization [1,12,13].

Techniques for localizing non-palpable breast tumors enable precise excision, helping surgeons achieve safe resection margins while preserving a maximum volume of healthy tissue [14]. The specimen plate radically improves orientation reliability, enhancing the efficacy of tumor localization and guiding accurate tumor removal, which in turn reduces re-excision rates and supports both oncologic control and cosmetic results. The specimen plate contributes to fewer re-excisions by providing unambiguous intraoperative mammograms for the surgeon and more precise radiologic evaluation of the specimen.

In our current study, the specimen plate was used specifically for the orientation of non-palpable or not clearly palpable breast cancers requiring preoperative localization. The molecular subtype of breast cancer significantly influences its detectability with different imaging modalities. It affects not only tumor visualization but also the choice and success of localization techniques. The effectiveness of preoperative tumor marking depends on both the imaging visibility and anatomical location of the lesion, which are influenced by tumor biology. Therefore, selecting an appropriate localization method—such as wire-guided, radio-guided, magnetic-based or other techniques—may require consideration of both the subtype and the spatial characteristics of the tumor [15,16,17].

Invasive lobular carcinomas (ILC) often grow in a diffuse, infiltrative pattern, making them difficult to detect with conventional imaging such as mammography or ultrasound. Similarly, triple-negative breast cancers (TNBCs) may lack calcifications or spiculated margins and can remain occult on mammography. Magnetic resonance imaging (MRI) has been shown to outperform other modalities in detecting both ILCs and TNBCs, offering the highest sensitivity across all techniques [18].

Breast cancer subtypes differ not only in biological behavior but also in imaging characteristics. Mammography is standard for detecting invasive NST carcinoma or in situ ductal carcinoma, especially through microcalcifications, but its sensitivity is reduced in dense breast tissue. Ultrasound performs better in dense breasts and in detecting ILCs, while MRI is particularly effective for multifocal lesions and aggressive subtypes such as TNBCs [18,19].

At our institution, intraoperative breast specimen verification is performed primarily with mammography and, less frequently, with ultrasound, depending on the radiological characteristics of the tumor. Tumor visibility on specimen mammography had a significant impact on the effectiveness of the specimen plate. Lesions with poor mammographic visibility posed greater orientation challenges, whereas well-visualized tumors were more easily aligned. Thus, imaging characteristics and molecular subtypes directly influenced the clinical utility of the specimen plate.

However, the highest proportion of non-R0 resections did not occur among the subtypes typically associated with poor imaging visibility and diffuse growth patterns, as described in the literature. This discrepancy may be attributed to the relatively small sample size and the fact that all cases involved non-palpable, small-sized tumors. Due to their limited volume and extent, the typical subtype-related differences in localization and margin clearance were less prominent in our cohort.

In our analysis, a substantial proportion of tumors with high proliferative activity were operated on following neoadjuvant therapy. As a result, the tumors encountered during specimen plate-guided orientation generally exhibited lower proliferative potential at the time of resection. Accordingly, the distribution of proliferation rates in the surgical specimens was similar across both study arms: in the suture marking group, 49 tumors (90.7%) showed low proliferation and 5 tumors (9.3%) showed high proliferation, while in the specimen plate group, 51 tumors (91.1%) showed low proliferation and 5 tumors (8.9%) showed high proliferation.

In our previous work, the specimen plate concept was introduced to address significant challenges—that were assessed by a nationwide questionnaire for the main breast cancer centers in Hungary—in breast specimen orientation during breast cancer surgery [5]. That study focused on the design and development of the plate, emphasizing its potential to improve orientation accuracy and reduce misinterpretation in the surgeon–pathologist–radiologist workflow.

In our study, tumor localization was recorded based on both the mammographic image and the photograph of the surgical specimen fixed on the specimen plate. The plate provided a fixed and reproducible reference frame, allowing consistent visual correlation between the specimen and the preoperative mammography. Although a formal grid system was not used, the combination of the specimen plate’s orientation and the imaging allowed precise documentation and evaluation of tumor location in all cases.

To ensure the internal validity of the study, we analyzed several key baseline characteristics across the two randomized groups. No significant differences were observed in patient age, body mass index (BMI), the proportion of patients receiving neoadjuvant therapy, or the proportion of tumors that became macroscopically detectable upon pathological assessment. Additionally, the distribution of histological subtypes, immunohistochemical profiles, tumor localization within the breast, and tumor size was comparable between the two arms, except for a higher pT0 rate in the Specimen Plate group. These similarities suggest that the two groups were clinically comparable, and that the differences observed in orientation performance and R0 resection rates can be attributed to the marking technique used, rather than initial group differences.

Jamaris et al. introduced a specimen orientation device that was developed specifically for non-palpable tumors following neoadjuvant therapy [20]. That device improved intraoperative specimen mammography by enabling more accurate radiologic presentation of the specimen, thereby contributing to a reduction in re-excision rates. However, that tool was designed for standard craniocaudal and mediolateral imaging planes and did not provide consistent support for anteroposterior or coronal plane visualization.

Unlike such devices, the specimen plate used in our study enables not only traditional mammographic projections but supports imaging in the coronal (frontal) plane. This orientation corresponds more closely to the surgeon’s intraoperative view and provides an intuitive spatial reference for both radiologists and pathologists. By offering this enhanced anatomical perspective, the specimen plate facilitates clearer interpretation of the margin status and tumor localization, especially in complex cases involving non-palpable lesions after neoadjuvant therapy.

The results from this randomized trial clearly demonstrate that the specimen plate not only enhances intraoperative handling but also provides superior mammographic images for assessing margins. Unlike suture marking, which can sometimes be ambiguous due to torsion or displacement of the specimen, the specimen plate offers consistent and noise-free safe orientation. The coronal plane images generated using the tool in question have proven to be easier for both surgeons and pathologists to interpret, as the breast scheme marked on the plate creates a visible X-ray shadow without obstructing the view, thanks to the plate’s radiolucent material.

Regarding tumor location, the anatomical quadrant within the breast did not influence the utility of the specimen plate. Instead, the intralesional position of the tumor within the excised specimen—particularly its proximity to the specimen margins—proved more relevant. When the tumor was located near one margin of the specimen, this direction could be reliably identified on intraoperative mammographic imaging using the plate. This allowed for targeted margin assessment and increased the likelihood of achieving clear margins, thereby reducing the need for reoperation.

Among patients receiving neoadjuvant therapy, a higher proportion of HER2-positive and triple-negative tumors was observed. Although these subtypes represent biologically heterogeneous forms of breast cancer, they tend to respond well to systemic neoadjuvant treatments. Furthermore, advances in early detection technologies—particularly in these subtypes—have contributed to the increasing prevalence of non-palpable tumors requiring image-guided localization and precise surgical orientation [21,22].

The literature reports re-excision rates in breast-conserving surgery of 4-30%, largely due to uncertainty about margin status. The superior accuracy of the specimen plate allows for evident identification of margins, contributing to fewer cases of ambiguous or positive margins. This not only benefits patient outcomes but may reduce the need for repeat surgeries, thus eventually improving overall patient safety and satisfaction.

The randomized study reveals that the additional time required for using the specimen plate was minimal, with only a few minutes added to the procedure, while the benefits in terms of improved orientation accuracy outweighed this minor inconvenience. Furthermore, informal feedback from participating surgeons and pathologists indicates a preference for the specimen plate over traditional suture marking, citing enhanced clarity in specimen orientation.

### Study Limitations

Although this study represents a significant step forward, some limitations should be acknowledged. The trial was conducted at a single institution, and while the results are promising, a multicentric study could further validate the findings. To address this, we are currently organizing a multicenter study to assess the applicability and effectiveness of the specimen plate across different institutions and clinical settings.

Due to the limited number of cases in each molecular subgroup, our findings remain exploratory, and an increased sample size along with a planned subsequent study will be necessary to determine which subtypes may benefit most from the use of the specimen plate. While the current number of cases limits the statistical power in several comparisons, the observed clinical tendencies suggest meaningful patterns that merit further investigation.

While our results indicate a reduction in re-excision rates when using the specimen plate, the long-term oncological implications of this improvement remain uncertain. It is yet to be determined whether enhanced specimen orientation translates into measurable benefits such as improved overall survival or disease-free survival. Ongoing follow-up of the study population, as well as future multicenter trials with extended observation periods, will be essential to evaluate these outcomes.

## 6. Conclusions

This study demonstrates that the specimen plate significantly improves orientation accuracy, mammographic clarity (80.4% vs. 13%, *p* = 0.0001), and the rate of successful R0 resections (91.1% vs. 77.8%, *p* = 0.067) in breast-conserving surgery. While its use requires minimal additional intraoperative time, the benefits in margin evaluation and re-excision reduction justify its implementation. As breast cancer treatment continues to evolve, tools like the Specimen Plate may play an important role in enhancing surgical precision and oncologic safety.

## 7. Patents

The process of obtaining design protection for the specimen plate described in this manuscript is currently underway.

## Figures and Tables

**Table 1:** Comparison of mean and median ages between groups (*p* = 0.5373).

Group	Mean Age (Years)	Median Age (Years)
Specimen Plate Group	61.1	64.0
Suture Marking Group	60.2	62.5

**Table 2:** Comparison of mean and median BMI between groups (*p* = 0.9888).

Group	Mean BMI (kg/m^2^)	Standard Deviation
Specimen Plate Group	26.23	3.55
Suture Marking Group	25.96	3.17

**Table 3:** Distribution of Histological Tumor Types by Specimen Orientation Method (*p* = 0.1734).

Group	NST Carcinoma	Lobular Carcinoma	Mucinous Carcinoma
Specimen Plate Group	91.07% (51 cases)	5.36% (3 cases)	3.57% (2 cases)
Suture Marking Group	87.04% (47 cases)	12.96% (7 cases)	0

**Table 4:** Distribution of immunohistochemical subtypes by specimen orientation method (*p* = 0.2208).

Group	HR+/HER2−	TNBC	TPBC	HR−/HER2+
Specimen Plate Group	78.57% (44 cases)	10.71% (6 cases)	5.36% (3 cases)	5.36% (3 cases)
Suture Marking Group	74.07% (40 cases)	12.96% (7 cases)	12.96% (7 cases)	0

**Table 5:** Tumor localization by breast quadrant according to specimen orientation technique (*p* = 0.6577).

Group	Upper Outer Quadrant	Upper Inner Quadrant	Lower Outer Quadrant	Lower Inner Quadrant	Central Quadrant
Specimen Plate Group	46.43% (26 cases)	21.43% (12 cases)	14.29% (8 cases)	12.50% (7 cases)	5.36% (3 cases)
Suture Marking Group	51.58% (28 cases)	20.37% (11 cases)	5.56% (3 cases)	14.81% (8 cases)	7.41% (4 cases)

**Table 6:** Distribution of tumor proliferative activity (Ki-67 Index) according to specimen orientation technique (*p* = 1.000).

Group	Low Proliferation	High Proliferation
Specimen Plate Group (n = 56)	91.1% (51 cases)	8.9% (5 cases)
Suture Marking Group (n = 54)	90.7% (49 cases)	9.3% (5 cases)

**Table 7:** Distribution of pathological tumor stage (pT) according to specimen orientation technique (*p* = 0.2050).

Group	T0	Tis	T1	T2	T3
Specimen Plate Group	16.07% (9 cases)	1.79% (1 case)	57.14% (32 cases)	23.21% (13 cases)	1.79% (1 case)
Suture Marking Group (n = 54)	5.56% (3 cases)	3.70% (2 cases)	72.22% (39 cases)	18.52% (10 cases)	0

**Table 8:** Comparison of Specimen Plate Group and Suture Marking Group.

Criteria	Specimen Plate Group (n = 56)	Suture Marking Group (n = 54)
Unambiguous orientation upon arrival in Pathology	100%	96.3% (52 cases)
Unambiguous tumor localization (macroscopic)	60.71% (34 cases)	44.44% (24 cases)
Evident laterality	100%	96.3% (52 cases)
Possibility of 180-degree rotation	0	0
Clear mammographic orientation	80.4% (45 cases)	13% (7 cases)
Successful R0 resection (*p* = 0.067)	91.1% (51 cases)	77.8% (42 cases)

**Table 9:** R0 and non-R0 resection rates by immunohistochemical subtype in the Specimen Plate Group.

Immunohistochemical Subtypes (n = 56)	R0 Resection (n = 52)	Non-R0 Resection (n = 4)
HR+/HER2−	(41 cases)	(3 cases)
TNBC	(5 cases)	(1 case)
TPBC	(3 cases)	(0 cases)
HR−/HER2+	(3 cases)	(0 cases)

**Table 10:** R0 and non-R0 resection rates by immunohistochemical subtype in the Suture Marking Group.

Immunohistochemical Subtypes (n = 54)	R0 Resection (n = 45)	Non-R0 Resection (n = 9)
HR+/HER2−	(31 cases)	(9 cases)
TNBC	(7 cases)	(0 cases)
TPBC	(7 cases)	(0 cases)

## Data Availability

Data are contained within the article.

## Abbreviations

The following abbreviations are used in this manuscript:

BMI	Body Mass Index
CC	Craniocaudal
DCIS	Ductal Carcinoma In Situ
ER	Estrogen Receptor
HER2	Human Epidermal Growth Factor Receptor 2
HR	Hormone Receptor
HR+/HER2−	Hormone Receptor-Positive/HER2-Negative
HR−/HER2+	Hormone Receptor-Negative/HER2-Positive
ILC	Invasive Lobular Carcinoma
MLO	Mediolateral Oblique
MRI	Magnetic Resonance Imaging
NST	No Special Type (Carcinoma)
pT	Pathological Tumor Stage
PR	Progesterone Receptor
R0	Resection with Negative Margins
TNBC	Triple-Negative Breast Cancer
TPBC	Triple-Positive Breast Cancer

## Appendix A

### Appendix A.1. Specimen Plate Manufacturing and Structural Features

The specimen plate is produced using 3D printing technology (e.g., a Creality Ender 5 printer) with thermoplastic polyurethane (TPU) A95 synthetic resin. This material provides a balance between flexibility and structural stability, allowing for secure fixation of the excised breast specimen using sutures, while maintaining sufficient elasticity to accommodate varying tissue consistency. TPU A95 ensures mechanical durability and retains its anchoring strength even when only a small surface area is used for fixation.

The 3D printing process uses a layer thickness of 0.1 mm, a printing temperature of 240 °C, and a nozzle diameter of 0.6 mm. The print speed is 30 mm/s. These settings create a fine, textile-like braided surface that supports firm yet adaptable suture anchoring.

The dimensions of the plate are 209 mm × 199.5 mm × 1.2 mm, sized to accommodate standard breast specimens while allowing for reproducible orientation. The design mimics the coronal plane anatomical layout of the female breast and axilla, enabling intuitive positioning of the specimen based on surgical anatomy.

### Appendix A.2. Radiographic and Ergonomic Properties

The plate is radiotranslucent, preventing interference with specimen mammography. It allows visualization of the tumor and surrounding tissue without introducing X-ray-positive artifacts, which supports precise radiologic–pathologic correlation. Unlike radiodense markers, the plate ensures that all internal structures remain clearly visible on mammographic images taken in the coronal plane.

By facilitating the consistent anatomical presentation of the specimen, the plate improves communication between surgeons, radiologists, and pathologists. It serves not only as a physical support but also as a visual reference system that enhances the reproducibility and interpretability of tumor localization and resection margin analysis.
